# Histogram analysis of multi-model high-resolution diffusion-weighted MRI in breast cancer: correlations with molecular prognostic factors and subtypes

**DOI:** 10.3389/fonc.2023.1139189

**Published:** 2023-04-28

**Authors:** Yanjin Qin, Feng Wu, Qilan Hu, Litong He, Min Huo, Caili Tang, Jingru Yi, Huiting Zhang, Ting Yin, Tao Ai

**Affiliations:** ^1^ Department of Radiology, Tongji Hospital, Tongji Medical College, Huazhong University of Science and Technology, Wuhan, China; ^2^ Department of Radiology, Xiangyang Central Hospital, Affiliated Hospital of Hubei University of Arts and Science, Xiangyang, China; ^3^ Department of Radiology, Xiantao First People’s Hospital Affiliated to Yangtze University, Xiantao, China; ^4^ Magnetic Resonance (MR) Scientific Marketing, Siemens Healthineers Ltd., Wuhan, China; ^5^ Magnetic Resonance (MR) Collaborations, Siemens Healthineers Ltd., Chengdu, China

**Keywords:** diffusion weight imaging, diffusion kurtosis imaging, intravoxel incoherent motion, breast cancer, prognosis, molecular subtypes

## Abstract

**Objective:**

To investigate the correlations between quantitative diffusion parameters and prognostic factors and molecular subtypes of breast cancer, based on a single fast high-resolution diffusion-weighted imaging (DWI) sequence with mono-exponential (Mono), intravoxel incoherent motion (IVIM), diffusion kurtosis imaging (DKI) models.

**Materials and Methods:**

A total of 143 patients with histopathologically verified breast cancer were included in this retrospective study. The multi-model DWI-derived parameters were quantitatively measured, including Mono-ADC, IVIM-*D*, IVIM-D*, IVIM-*f*, DKI-Dapp, and DKI-Kapp. In addition, the morphologic characteristics of the lesions (shape, margin, and internal signal characteristics) were visually assessed on DWI images. Next, Kolmogorov–Smirnov test, Mann-Whitney *U* test, Spearman’s rank correlation, logistic regression, receiver operating characteristic (ROC) curve, and Chi-squared test were utilized for statistical evaluations.

**Results:**

The histogram metrics of Mono-ADC, IVIM-*D*, DKI-Dapp, and DKI-Kapp were significantly different between estrogen receptor (ER)-positive *vs*. ER-negative groups, progesterone receptor (PR)-positive *vs*. PR-negative groups, Luminal *vs*. non-Luminal subtypes, and human epidermal receptor factor-2 (HER2)-positive *vs*. non-HER2-positive subtypes. The histogram metrics of Mono-ADC, DKI-Dapp, and DKI-Kapp were also significantly different between triple-negative (TN) *vs*. non-TN subtypes. The ROC analysis revealed that the area under the curve considerably improved when the three diffusion models were combined compared with every single model, except for distinguishing lymph node metastasis (LNM) status. For the morphologic characteristics of the tumor, the margin showed substantial differences between ER-positive and ER-negative groups.

**Conclusions:**

Quantitative multi-model analysis of DWI showed improved diagnostic performance for determining the prognostic factors and molecular subtypes of breast lesions. The morphologic characteristics obtained from high-resolution DWI can be identifying ER statuses of breast cancer.

## Introduction

Diffusion-weighted imaging (DWI) has been proven to be a potential diagnostic tool for the evaluation of breast lesions (1). In clinical practice, single-shot echo planar imaging (ss-EPI) sequence is commonly utilized in breast DWI protocols due to fast acquisition time (2, 3). However, previous studies have seldom evaluated the morphologic analysis of lesions on ss-EPI images due to geometric distortion and poor spatial resolution (4, 5). Readout-segmented EPI (rs-EPI) can reduce distortions and maintain high resolution, but it was limited by long scan times (6). Recently, the simultaneous multislice (SMS) technique, which allows the exciting acquire multiple slices at once, has been introduced to reduce the scan time (7, 8). The SMS technique was combined with rs-EPI to generate images with less image distortion and higher spatial resolution for breast lesions in a clinically acceptable scanning duration (9, 10). The application of SMS rs-EPI makes it feasible to qualitatively assess the morphologic characteristics of breast lesions.

In addition to the qualitative analysis of SMS rs-EPI images, our study also focused on the quantitative analysis of multi-model DWI. Conventional DWI is based on a mono-exponential (Mono) model that was first proposed to reflect the random Brownian motion of water molecules diffusing into biological media by quantifying apparent diffusion coefficient (ADC) values (11). However, water diffusion in complex biological media may be influenced by the blood microcirculation in capillaries, leading to a non-Gaussian distribution (12, 13). To address this, advanced diffusion models, including intravoxel incoherent motion (IVIM) and diffusion kurtosis imaging (DKI), have been developed to reflect the diffusion behavior of water molecules in tumors more accurately (14). A few studies have investigated the correlations between IVIM- or DKI-derived parameters with several clinical prognostic factors and molecular subtypes. However, the conclusions have still not reached a consensus (15, 16). Furthermore, most studies have reported that more information can be parsed from histogram analysis, which can reflect the microstructures and heterogeneity of breast cancer (17–20).

Therefore, the aim of this study was to apply three diffusion models (Mono, IVIM, and DKI) to determine the parameters valuable for differentiating between prognostic factor statuses and molecular subtypes, as well as to assess the correlations of morphologic characteristics with prognostic factors and molecular subtypes.

## Materials and methods

### Patients

This retrospective study was approved by our institutional review board, and informed consent was obtained. From September 2020 to May 2021, 216 female patients who underwent breast MRI in our hospital and fulfilled the following criteria were selected: (1) the patients did not undergo chemotherapy, or any other interventions before they were examined by MRI; (2) the pathologic type of breast lesions was confirmed by surgery or biopsy; and (3) relevant pathologic data of patients were complete. The exclusion criteria included: 1) non-mass like enhancement lesions detected on dynamic contrast-enhanced (DCE)-MRI (n = 34); 2) the max diameter of mass lesions< 1cm (n = 16); 3) poor DWI image quality due to patient motion or susceptibility artifact (n = 23). Only the largest lesion was analyzed when multiple lesions were detected in the bilateral breast. Finally, 143 patients (mean age, 48.57 ± 12.01 years, range, 26 – 81 years) with 143 mass lesions (mean diameter, 2.48 ± 0.95 cm) were included in the study. More detailed characteristics of the 143 patients are summarized in **Table 1**.

**Table 1:** Study population and histopathological characteristics.

Characteristics		N (%)
Age at diagnosis	≤ 50	78 (54.5)
	> 50	65 (45.5)
Long diameter (cm)	> 2	91 (63.6)
	≤ 2	52 (36.4)
Side	Right	68 (47.6)
	Left	75 (52.4)
Menopausal status	Premenopausal	74 (51.7)
	Postmenopausal	69 (48.3)
Histological type	IDC	109 (76.2)
	ILC	11 (7.7)
	Papillary carcinoma	8 (5.6)
	DCIS	15 (10.5)
ER	Positive	80 (55.9)
	Negative	63 (44.1)
PR	Positive	76 (53.1)
	Negative	67 (46.9)
HER2	Positive	48 (33.6)
	Negative	95 (66.4)
Ki-67	Positive	79 (55.2)
	Negative	64 (44.8)
LNM	Positive	49 (34.3)
	Negative	94 (65.7)
Molecular subtypes		
Luminal A		45 (31.5)
Luminal B		41 (28.7)
HER2-positive		25 (17.5)
Triple-negative		32 (22.3)
Morphological features		
Shape	Round	24 (16.8)
	Oval	49 (34.3)
	Irregular	70 (48.9)
Margin	Smooth	76 (53.1)
	Spiculated	16 (11.2)
	Irregular	51 (35.7)
Signal	Homogenous	55 (38.4)
	Heterogenous	64 (44.8)
	Rim	24 (16.8)

DCIS, Ductal carcinoma in situ; IDC, Invasive ductal carcinoma; ILC, Invasive lobular carcinoma; ER, Estrogen receptor; PR, Progesterone receptor; HER2, Human epidermal growth factor receptor 2; LNM, Lymph nodes metastasis; TN, Triple-negative.

### MRI scans

Breast MRI was performed on a 3T MRI scanner (MAGNETOM Skyra, Siemens Healthcare, Erlangen, Germany) using a dedicated 16-channel phased-array bilateral breast surface coil. The breast MRI protocol included the following sequences: axial fat-saturated T2-weighted imaging, T1-weighted DCE-MRI with the time-resolved angiography (TWIST) with a volumetric interpolated breath-hold examination (VIBE) technique, and SMS rs-EPI sequence. Detailed imaging parameters are provided in **Table 2**.

**Table 2:** Sequence parameters for T2-weighted imaging, SMS rs-EPI, and DCE-MRI.

Parameters	T2WI	SMS rs-EPI	DCE-MRI
Repetition time (ms)	3700	2350	5.24
Echo time (ms)	101	72	2.46
Field of view (mm^2^)	320 x 320	280 x 280	320 x 320
Matrix	224 x 320	122 x 188	182 x 320
Slice thickness (mm)	4	5	1.5
Pixel bandwidth (Hz/Px)	347	887	780
Parallel imaging	GRAPPA (x2)	GRAPPA (x2)	CAIPIRINHA (x4)
b-values (sec/mm^2^)	/	0, 50, 100, 200, 400, 800, 1000, 2000	/
Readout segment	/	5	/
Multi-slice mode	/	Slice acceleration (x2)	/
Temporal resolution (sec/phase)	/	/	5.74
Acquisition time (min:sec)	2:06	4:39	5:57

SMS, Simultaneous multi-slice; DCE, Dynamic contrast-enhanced; GRAPPA, Generalized autocalibrating partially parallel acquisition; CAIPIRINHA, Controlled aliasing in parallel imaging results in higher acceleration.

/ indicates Non-applicable.

### Image analysis

The images were independently analyzed by two breast readers (with 3 and 5 years of experience, respectively) using an in-house-developed DKI tool software. Both readers were informed that the patients had breast cancer but were blinded to the detailed pathologic data. Two-dimensional (2D) region of interest (ROI) were manually delineated, which excluded the cystic or necrotic portions of the tumor, on high-*b*-value (*b*=1000 s/mm^2^) SMS rs-EPI images, with the reference of the corresponding T2-weighted and DCE-MRI images. The ROI was then copied to other parametric maps [including ADC, pure diffusion (*D*), pseudo-diffusion coefficient (D*), perfusion fraction (*f*), apparent diffusional kurtosis (Kapp), and apparent diffusion coefficient (Dapp) maps] using the DKI tool software. Finally, the histogram information of each ROI map was generated, including mean, median, percentile values (25th and 75th), kurtosis, and skewness. For example, the mean and 75th percentile metrics of Mono-ADC were presented as Mono-ADC_mean_ and Mono-ADC_75th_, respectively.

The corresponding mathematical expressions were as follows:

1. Mono-exponential model (16):


Sb/S0=exp (−bADC)

where *S*_b_ is the signal intensity on the DWI image at a certain *b* value (800 sec/mm^2^) and *S*_0_ is the signal intensity value in the voxels with *b* values of 0.

2.DKI model (21):


$$\ln[S(b)/S0]=-bD_{app}+\frac{1}{6}b^{2}D_{app}{\;}^{2}K_{app}$$

where *S*_b_ is he signal intensity on the DWI image according to all b-values (0, 50, 100, 200, 400, 800, 1000, and 2000 sec/mm^2^). D_app_ represents the non-Gaussian diffusion coefficient and K_app_ represents the apparent kurtosis coefficient without unit.

3. IVIM model (22):


$$S_{b}/S_{0}=(1-f)\exp(-bD)+fexp[-b(D*+D)]$$

where *S*_b_ is he signal intensity on the DWI image according to the b-value (0, 50, 100, 200, and 400 sec/mm^2^). *D* is the true diffusion coefficient representing the simple movement of water molecules in the tissue (unit: mm^2^/s), D* is the pseudo-diffusion coefficient representing perfusion-related diffusion (unit: mm^2^/s), and *f* is the fraction of fast diffusion representing the diffusion linked to microcirculation (0 ≤ *f* ≤ 1).

### Morphologic analysis

Two experienced readers independently assessed several morphologic characteristics on SMS rs-EPI images with *b* = 1000 mm^2^/s according to the Breast Imaging Reporting and Data System lexicon (BI-RADS edition 2013). Since enhancement is mostly used to evaluate breast lesions on DCE-MRI, two readers evaluated breast lesions on DWI images using internal signal characteristics, which were defined as lesions with homogeneous signal, heterogeneous signal, or only high-signal at the rim (23, 24). Each morphological characteristic was specifically evaluated as follows:

- Lesion shape: 1 for round, 2 for oval, 3 for irregular.
- Lesion margin: 1 for smooth, 2 for spiculated, 3 for irregular.
- Lesion internal signal characteristics: 1 for heterogeneous, 2 for homogeneous, 3 for rim.
- The max diameter of lesion was measured on the largest tumor section.

### Histopathologic assessment

Histopathologic results were obtained from the electronic medical records of each patient in our hospital. Estrogen receptor (ER) positivity and progesterone receptor (PR) positivity were defined as the presence of 1% or more positively stained nuclei in 10 high-power fields (25). Human epidermal growth factor receptor 2 (HER2) was considered positive if it was scored 3+ for immunohistochemically stained tissue, or gene amplification was observed with fluorescence *in situ* hybridization (FISH) (26). More than 20% of cancer nuclei were positively stained for Ki-67 (12). Lymph node metastasis (LNM) was confirmed by the clinician performing the histopathologic examination (13). According to the statuses of ER, PR, HER2, and Ki-67, the breast tumors were further classified as Luminal A, Luminal B, HER2-positive, and triple-negative (TN) (27).

### Statistical analysis

All statistical analyses were performed using MedCalc software (version 15.0, Ostend, Belgium) and SPSS software (version 26.0, IL, USA). The inter-reader agreement for diffusion parameters and morphological characteristics was assessed by using the intraclass correlation coefficient (ICC): ICC ≤ 0.40, poor agreement; 0.40 - 0.59, fair agreement; 0.60 - 0.74, good agreement; 0.75 - 1.00, excellent agreement. The categorical variables were as follows: prognostic factors including ER, PR, HER2, Ki-67, and LNM (positive *vs*. negative) and molecular subtypes (Luminal type *vs*. non-Luminal type, TN type *vs*. non-TN type, and HER2-positive type *vs*. non-HER2-positive type). All data were tested first with the Kolmogorov–Smirnov test for normality analysis. The quantitative diffusion parameters and max diameter of lesions between different subgroups were compared using the Mann–Whitney *U* test. Spearman correlations were used to characterize the correlations of multi-model-derived histogram metrics with prognostic factors and molecular subtypes. With pathologic results as the gold standard, the receiver operating characteristic (ROC) curve analysis was used to assess the diagnostic efficacy of each parameter or each model, and the area under the ROC curve (AUC) was calculated. Then, the largest AUC of each parameter was selected to establish the IVIM model (*D*, D*, and *f*), the DKI model (Kapp and Dapp), and the combined three diffusion models (Mono, IVIM, and DKI) using logistic regression. The AUC comparisons were performed using the DeLong test. The morphologic characteristics were compared using the Chi-squared test. For all tests, the significance was set at *p*< 0.05/8 = 0.00625 (control for multiple comparisons across five prognostic factors and three molecular subtypes).

## Results

### Inter-reader agreement

As shown in **Table 3**, there was an excellent agreement between two readers regarding the representative mean and median metrics of diffusion parameters (range of ICCs, 0.827 – 0.939) and morphological characteristics including the shape, margin, and internal signal (range of ICCs, 0.857 – 0.890).

**Table 3:** Interobserver agreement for diffusion parameters and morphological characteristics by two readers.

Parameters	Metrics	ICC	95% Confidence Interval
Mono-ADC	mean	0.893	0.854 – 0.922
	median	0.882	0.839 – 0.914
IVIM-*D*	mean	0.861	0.807 – 0.900
	median	0.827	0.759 – 0.875
IVIM-D*	mean	0.939	0.915 – 0.956
	median	0.918	0.885 – 0.941
IVIM-*f*	mean	0.832	0.766 – 0.879
	median	0.871	0.820 – 0.907
DKI-Kapp	mean	0.889	0.849 – 0.919
	median	0.933	0.908 – 0.951
DKI-Dapp	mean	0.927	0.900 – 0.947
	median	0.918	0.888 – 0.940
Morphological characteristics			
Shape		0.857	0.807 – 0.895
Margin		0.867	0.819 – 0.902
Internal signal		0.890	0.851 – 0.920

ICC, intraclass correlation coefficient. D* is pseudo-diffusion coefficient.

### Relationship of diffusion parameters with prognostic factors and molecular subtypes

The histogram metrics of various diffusion parameters among prognostic factors and molecular subtypes of breast cancer are displayed in **Table 4**. For Mono-ADC, IVIM-*D*, and DKI-Dapp, all histogram metrics (mean, median, 25th, and 75th percentile) were significantly lower while DKI-Kapp histogram metrics were significantly higher in ER-positive groups compared to those in ER-negative groups (all *p*< 0.0625), the same trend was found in PR-positive groups compared with the PR-negative groups (all *p*< 0.0625).

**Table 4:** Comparisons of mono, IVIM and DKI histogram metrics between different groups with molecular prognostic factors and subtypes.

		ER		PR		HER2		Ki-67	
Parameters	Histogram metrics	Negative	Positive	Negative	Positive	Negative	Positive	Negative	Positive
Mono-ADC	25th	**0.869 ± 0.147**	**0.739 ± 0.118**	**0.853 ± 0.149**	**0.746 ± 0.125**	0.789 ± 0.148	0.809 ± 0.144	0.788 ± 0.135	0.803 ± 0.156
	Median	**0.963 ± 0.163**	**0.812 ± 0.122**	**0.948 ± 0.163**	**0.817 ± 0.130**	0.867 ± 0.160	0.902 ± 0.159	0.868 ± 0.144	0.888 ± 0.172
	Mean	**0.979 ± 0.163**	**0.827 ± 0.126**	**0.964 ± 0.165**	**0.832 ± 0.131**	0.880 ± 0.161	0.921 ± 0.160	0.881 ± 0.145	0.904 ± 0.174
	75th	**1.073 ± 0.192**	**0.906 ± 0.141**	**1.060 ± 0.192**	**0.909 ± 0.146**	0.960 ± 0.181	1.018 ± 0.187	0.964 ± 0.168	0.991 ± 0.198
	Kurtosis	3.739 ± 1.815	3.650 ± 1.859	3.615 ± 1.783	3.755 ± 1.887	3.903 ± 2.001	3.266 ± 1.370	3.597 ± 1.708	3.764 ± 1.937
	Skewness	0.612 ± 0.676	0.509 ± 0.717	0.615 ± 0.633	0.500 ± 0.752	0.582 ± 0.741	0.499 ± 0.610	0.453 ± 0.764	0.636 ± 0.634
IVIM-*D*	25th	**0.990 ± 0.234**	**0.857 ± 0.205**	**0.971 ± 0.231**	**0.867 ± 0.214**	0.914 ± 0.229	0.919 ± 0.228	0.909 ± 0.230	0.921 ± 0.227
	Median	**1.128 ± 0.226**	**0.994 ± 0.174**	**1.113 ± 0.226**	**1.000 ± 0.178**	1.044 ± 0.210	1.072 ± 0.207	1.041 ± 0.189	1.063 ± 0.224
	Mean	**1.147 ± 0.223**	**1.008 ± 0.168**	**1.131 ± 0.224**	**1.016 ± 0.172**	1.059 ± 0.205	1.091 ± 0.206	1.056 ± 0.181	1.080 ± 0.224
	75th	**1.280 ± 0.238**	**1.136 ± 0.185**	**1.266 ± 0.241**	**1.141 ± 0.184**	1.182 ± 0.214	1.235 ± 0.232	1.183 ± 0.190	1.213 ± 0.243
	Kurtosis	3.440 ± 1.553	3.349 ± 1.366	3.376 ± 1.519	3.401 ± 1.390	3.585 ± 1.613	3.002 ± 0.944	3.416 ± 1.583	3.367 ± 1.337
	Skewness	0.459 ± 0.628	0.390 ± 0.608	0.481 ± 0.597	0.366 ± 0.631	0.465 ± 0.650	0.331 ± 0.537	0.328 ± 0.682	0.495 ± 0.550
IVIM-D*	25th	3.211 ± 4.432	2.775 ± 3.992	2.884 ± 3.961	3.040 ± 4.393	3.188 ± 4.477	2.530 ± 3.531	3.292 ± 4.543	2.704 ± 3.875
	Median	8.321 ± 5.900	7.307 ± 5.040	7.857 ± 5.536	7.663 ± 5.387	7.818 ± 5.462	7.627 ± 5.448	8.050 ± 5.501	7.513 ± 5.412
	Mean	10.022 ± 4.871	9.096 ± 4.040	9.533 ± 4.460	9.478 ± 4.439	9.583 ± 4.435	9.347 ± 4.472	9.741 ± 4.719	9.312 ± 4.208
	75th	14.621 ± 6.304	13.424 ± 5.334	14.180 ± 6.017	13.749 ± 5.617	14.020 ± 5.387	13.816 ± 6.576	13.963 ± 6.027	13.941 ± 5.632
	Kurtosis	5.959 ± 9.492	5.912 ± 4.516	5.874 ± 5.417	5.984 ± 4.538	5.868 ± 5.174	6.060 ± 4.527	5.445 ± 3.703	6.327 ± 5.762
	Skewness	1.282 ± 1.001	1.278 ± 0.907	1.256 ± 1.004	1.301 ± 0.898	1.236 ± 0.961	1.367 ± 0.921	6.327 ± 5.762	1.333 ± 1.029
IVIM-*f*	25th	0.027 ± 0.025	0.025 ± 0.023	0.026 ± 0.025	0.026 ± 0.025	0.027 ± 0.025	0.024 ± 0.021	0.026 ± 0.024	0.026 ± 0.023
	Median	0.052 ± 0.029	0.051 ± 0.031	0.052 ± 0.029	0.052 ± 0.032	0.052 ± 0.032	0.052 ± 0.027	0.055 ± 0.033	0.050 ± 0.029
	Mean	0.058 ± 0.028	0.057 ± 0.030	0.057 ± 0.028	0.058 ± 0.031	0.057 ± 0.031	0.059 ± 0.027	0.061 ± 0.032	0.055 ± 0.027
	75th	0.082 ± 0.041	0.081 ± 0.046	0.082 ± 0.040	0.082 ± 0.047	0.080 ± 0.046	0.084 ± 0.041	0.087 ± 0.049	0.077 ± 0.039
	Kurtosis	3.695 ± 2.332	3.597 ± 2.970	3.533 ± 2.255	3.735 ± 3.049	3.814 ± 3.168	3.296 ± 1.327	3.388 ± 1.720	3.845 ± 3.283
	Skewness	0.626 ± 0.709	0.624 ± 0.758	0.591 ± 0.702	0.654 ± 0.765	0.662 ± 0.786	0.551 ± 0.619	0.611 ± 0.643	0.635 ± 0.804
DKI-Kapp	25th	**0.796 ± 0.128**	**0.901 ± 0.158**	**0.811 ± 0.126**	**0.893 ± 0.167**	0.872 ± 0.159	0.820 ± 0.139	0.867 ± 0.142	0.844 ± 0.163
	Median	**0.883 ± 0.125**	**0.993 ± 0.165**	**0.895 ± 0.126**	**0.988 ± 0.171**	0.956 ±0.168	0.921 ± 0.136	0.966 ± 0.137	0.927 ± 0.173
	Mean	**0.878 ± 0.124**	**0.991 ± 0.148**	**0.890 ± 0.122**	**0.986 ± 0.155**	0.956 ± 0.151	0.911 ± 0.140	0.957 ± 0.144	0.928 ± 0.151
	75th	**0.963 ± 0.133**	**1.081 ± 0.160**	**0.973 ± 0.128**	**1.078 ± 0.169**	1.037 ± 0.164	1.013 ± 0.149	1.051 ± 0.154	1.011 ± 0.162
	Kurtosis	3.594 ± 2.031	3.653 ± 1.885	3.618 ± 2.153	3.635 ± 1.753	3.751 ± 1.909	3.381 ± 2.009	3.567 ± 1.765	3.675 ± 2.087
	Skewness	-0.124 ± 0.752	0.005 ± 0.812	-0.136 ± 0.742	0.023 ± 0.821	0.021 ± 0.804	-0.195 ± 0.738	-0.046 ± 0.813	-0.056 ± 0.769
DKI-Dapp	25th	**1.179 ± 0.209**	**1.003 ± 0.208**	**1.148 ± 0.211**	**1.014 ± 0.217**	1.072 ± 0.238	1.087 ± 0.194	1.079 ± 0.206	1.075 ± 0.239
	Median	**1.318 ± 0.227**	**1.124 ± 0.203**	**1.298 ± 0.227**	**1.131 ± 0.213**	1.195 ± 0.241	1.238 ± 0.219	1.205 ± 0.217	1.213 ± 0.249
	Mean	**1.335 ± 0.223**	**1.146 ± 0.205**	**1.316 ± 0.225**	**1.153 ± 0.211**	1.215 ± 0.241	1.259 ± 0.211	1.222 ± 0.217	1.236 ± 0.245
	75th	**1.481 ± 0.265**	**1.274 ± 0.220**	**1.467 ± 0.265**	**1.276 ± 0.224**	1.339 ± 0.262	1.417 ± 0.256	1.352 ± 0.245	1.377 ± 0.275
	Kurtosis	3.469 ± 1.641	3.311 ± 1.160	3.314 ± 1.575	3.439 ± 1.210	3.552 ± 1.528	3.041 ± 0.992	3.419 ± 1.243	3.349 ± 1.505
	Skewness	0.503 ± 0.671	0.471 ± 0.604	0.507 ± 0.602	0.466 ± 0.661	0.540 ± 0.657	0.377 ± 0.571	0.387 ± 0.673	0.562 ± 0.590
		LNM		Luminal		TN		HER2	
Parameters	Histogram metrics	Negative	Positive	Non-Luminal	Luminal	Non-TN	TN	Non-HER2-positive	HER2-positive
Mono-ADC	25th	0.805 ± 0.151	0.780 ± 0.137	**0.878 ± 0.142**	**0.742 ± 0.123**	**0.772 ± 0.139**	**0.878 ± 0.143**	**0.779 ± 0.142**	**0.877 ± 0.144**
	Median	0.885 ± 0.165	0.867 ± 0.150	**0.975 ± 0.159**	**0.815 ± 0.126**	**0.853 ± 0.150**	**0.967 ± 0.163**	**0.856 ± 0.152**	**0.984 ± 0.155**
	Mean	0.903 ± 0.167	0.877 ± 0.150	**0.991 ± 0.160**	**0.830 ± 0.128**	**0.869 ± 0.152**	**0.982 ± 0.165**	**0.871 ± 0.154**	**1.002 ± 0.155**
	75th	0.986 ± 0.192	0.966 ± 0.172	**1.087 ± 0.190**	**0.908 ± 0.143**	**0.953 ± 0.174**	**1.071 ± 0.196**	**0.952 ± 0.174**	**1.107 ± 0.184**
	Kurtosis	3.726 ± 1.954	4.084 ± 2.561	3.733 ± 1.897	3.660 ± 1.802	3.645 ± 1.780	3.842 ± 2.032	3.709 ± 1.860	3.593 ± 1.738
	Skewness	0.645 ± 0.635	0.380 ± 0.785	0.616 ± 0.667	0.513 ± 0.720	0.525 ± 0.704	0.657 ± 0.680	0.552 ± 0.709	0.564 ± 0.661
IVIM-*D*	25th	0.920 ± 0.249	0.907 ± 0.182	**0.996 ± 0.236**	**0.862 ± 0.206**	0.897 ± 0.210	0.981 ± 0.273	0.895 ± 0.231	1.016 ± 0.181
	Median	1.057 ± 0.225	1.046 ± 0.174	**1.138 ± 0.227**	**0.997 ± 0.175**	1.034 ± 0.195	1.120 ± 0.240	**1.030 ± 0.201**	**1.160 ± 0.212**
	Mean	1.073 ± 0.220	1.063 ± 0.177	**1.158 ± 0.224**	**1.011 ± 0.169**	1.049 ± 0.189	1.142 ± 0.242	**1.047 ± 0.199**	**1.178 ± 0.202**
	75th	1.201 ± 0.232	1.198 ± 0.199	**1.294 ± 0.239**	**1.137 ± 0.184**	1.178 ± 0.209	1.276 ± 0.248	**1.174 ± 0.211**	**1.319 ± 0.231**
	Kurtosis	3.437 ± 1.540	3.296 ± 1.259	3.368 ± 1.521	3.403 ± 1.405	3.323 ± 1.354	3.617 ± 1.737	3.461 ± 1.497	3.049 ± 1.145
	Skewness	0.429 ± 0.614	0.403 ± 0.625	0.470 ± 0.590	0.387 ± 0.633	0.394 ± 0.613	0.513 ± 0.628	0.421 ± 0.632	0.416 ± 0.547
IVIM-D*	25th	3.278 ± 4.341	2.371 ± 3.831	3.033 ± 4.076	2.923 ± 4.275	2.779 ± 4.028	3.619 ± 4.689	3.112 ± 4.381	2.282 ± 3.055
	Median	8.010 ± 5.551	7.262 ± 5.238	8.117 ± 5.747	7.513 ± 5.246	7.642 ± 5.281	8.140 ± 6.029	7.683 ± 5.450	8.088 ± 5.488
	Mean	9.706 ± 4.516	9.116 ± 4.289	9.726 ± 4.687	9.356 ± 4.278	9.356 ± 4.298	10.015 ± 4.913	9.535 ± 4.447	9.357 ± 4.454
	75th	14.146 ± 5.909	13.578 ± 5.599	14.291 ± 6.284	13.736 ± 5.466	13.732 ± 5.710	14.712 ± 6.095	13.994 ± 5.634	13.752 ± 6.604
	Kurtosis	6.106 ± 5.296	5.600 ± 4.243	5.984 ± 5.763	5.899 ± 4.367	6.014 ± 4.668	5.648 ± 5.904	5.831 ± 4.806	6.412 ± 5.668
	Skewness	1.287 ± 0.998	1.267 ± 0.847	1.270 ± 1.049	1.287 ± 0.878	1.306 ± 0.933	1.191 ± 1.002	1.261 ± 0.910	1.371 ± 1.118
IVIM-*f*	25th	0.027 ± 0.023	0.023 ± 0.025	0.027 ± 0.025	0.025 ± 0.023	0.025 ± 0.023	0.029 ± 0.028	0.026 ± 0.024	0.024 ± 0.022
	Median	0.053 ± 0.030	0.049 ± 0.032	0.053 ± 0.030	0.051 ± 0.031	0.051 ± 0.030	0.055 ± 0.034	0.052 ± 0.032	0.050 ± 0.025
	Mean	0.059 ± 0.030	0.055 ± 0.029	0.059 ± 0.029	0.057 ± 0.030	0.056 ± 0.028	0.062 ± 0.035	0.058 ± 0.031	0.054 ± 0.019
	75th	0.083 ± 0.045	0.079 ± 0.042	0.084 ± 0.042	0.080 ± 0.045	0.080 ± 0.041	0.088 ± 0.052	0.082 ± .047	0.078 ± 0.024
	Kurtosis	3.719 ± 3.064	3.490 ± 1.825	3.597 ± 2.395	3.669 ± 2.896	3.570 ± 2.628	3.882 ± 2.965	3.727 ± 2.904	3.231 ± 1.335
	Skewness	0.618 ± 0.785	0.638 ± 0.632	0.591 ± 0.744	0.647 ± 0.731	0.607 ± 0.713	0.684 ± 0.814	0.657 ± 0.751	0.471 ± 0.641
DKI-Kapp	25th	0.846 ± 0.169	0.871 ± 0.119	**0.794 ± 0.124**	**0.895 ± 0.159**	**0.867 ± 0.164**	**0.813 ± 0.106**	**0.873 ± 0.151**	**0.769 ± 0.141**
	Median	0.936 ± 0.170	0.960 ± 0.133	**0.879 ± 0.125**	**0.988 ± 0.164**	**0.961 ± 0.164**	**0.887 ± 0.121**	**0.961 ± 0.159**	**0.867 ± 0.131**
	Mean	0.932 ± 0.156	0.957 ± 0.132	**0.873 ± 0.119**	**0.986 ± 0.149**	**0.957 ± 0.155**	**0.886 ± 0.105**	**0.959 ± 0.145**	**0.856 ± 0.136**
	75th	1.020 ± 0.167	1.047 ± 0.144	**0.956 ± 0.127**	**1.077 ± 0.161**	**1.049 ± 0.165**	**0.961 ± 0.117**	1.046 ± 0.158	0.950 ± 0.141
	Kurtosis	3.388 ± 1.489	4.084 ± 2.561	3.738 ± 2.298	3.553 ± 1.679	3.629 ± 1.951	3.619 ± 1.951	3.571 ± 1.749	3.891 ± 2.713
	Skewness	-0.068 ± 0.708	-0.020 ± 0.926	-0.151 ± 0.789	0.015 ± 0.782	-0.060 ± 0.801	-0.023 ± 0.744	0.001 ± 0.769	-0.315 ± 0.830
DKI-Dapp	25th	1.084 ± 0.235	1.062 ± 0.201	**1.185 ±0.197**	**1.005 ± 0.212**	**1.044 ± 0.217**	**1.192 ± 0.211**	**1.056 ± 0.227**	**1.176 ± 0.182**
	Median	1.214 ±0.243	1.201 ± 0.219	**1.335 ±0.218**	**1.126 ± 0.207**	**1.176 ± 0.225**	**1.326 ± 0.230**	**1.180 ± 0.230**	**1.345 ± 0.205**
	Mean	1.238 ± 0.242	1.214 ± 0.213	**1.352 ± 0.216**	**1.148 ± 0.206**	**1.196 ± 0.221**	**1.347 ± 0.234**	**1.202 ± 0.231**	**1.359 ± 0.195**
	75th	1.371 ± 0.270	1.354 ± 0.246	**1.503 ± 0.261**	**1.275 ±0.220**	**1.331 ± 0.249**	**1.483 ± 0.272**	**1.331 ± 0.252**	**1.527 ± 0.250**
	Kurtosis	3.458 ± 1.483	3.231 ± 1.189	3.414 ± 1.674	3.358 ± 1.174	3.290 ± 1.182	3.694 ± 1.937	3.449 ± 1.420	3.057 ± 1.026
	Skewness	0.563 ± 0.622	0.337 ± 0.631	0.500 ± 0.636	0.476 ± 0.633	0.444 ± 0.623	0.628 ± 0.654	0.517 ± 0.639	0.336 ± 0.585

The data for significance is shown in bold (p < 0.0625). ER, Estrogen receptor; PR, Progesterone receptor; HER2, Human epidermal growth factor receptor 2; LNM, Lymph nodes metastasis; TN, Triple-negative.

Luminal type *vs*. non-Luminal type revealed that considerable differences originated from histogram metrics (mean, median, 25th, and 75th percentile) of Mono-ADC, IVIM-*D*, DKI-Kapp, and DKI-Dapp (all *p*< 0.0625). Significantly higher histogram metrics (mean, median, 25th, and 75th percentile) of Mono-ADC and DKI-Dapp while lower histogram metrics (mean, median, 25th, and 75th percentile) of DKI-Kapp were found in the TN type than in the non-TN type (all p< 0.0625). Additionally, the Mono-ADC (mean, median, 25th, and 75th percentile), IVIM-*D*(mean, median, and 75th percentile), and DKI-Dapp (mean, median, 25th, and 75th percentile) values were significantly higher and the DKI-Kapp (mean, median, and 25th percentile) values were significantly lower in the HER2-positive type than in the non-HER2-positive type (all *p*< 0.0625). No statistically significant difference was observed in the negative and positive groups between HER2, Ki-67, and LNM (all *p* > 0.00625).

**Figure 1:**
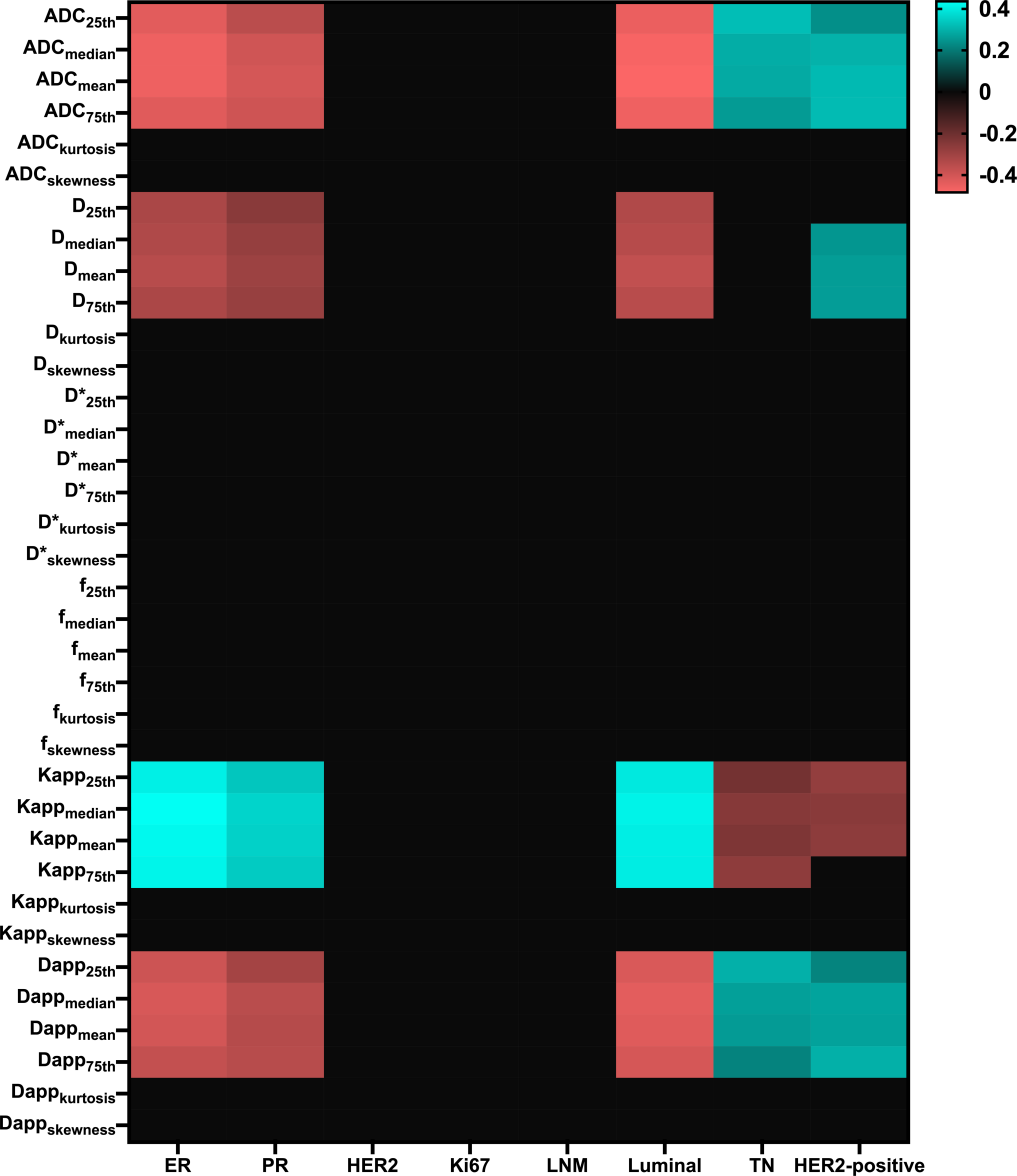
Matrix plot of the Pearson correlation coefficients between multi-model histogram parameters with molecular prognostic factors and subtypes. Colored entries indicate significant correlations (p<0.0625) with positive (blue) or negative (red). ER, Estrogen receptor; PR, Progesterone receptor; HER2, Human epidermal growth factor receptor 2; LNM, Lymph nodes metastasis; TN, Triple-negative.; D*, pseudo-diffusion coefficient.

Considerable correlations were observed between ER and PR groups as well as Luminal, TN, and HER2-positive types. Diffusion parameters (Mono-ADC, IVIM-*D*, DKI-Dapp, and DKI-Kapp) largely involved the histogram metrics (mean, median, 25th, and 75th percentile). When including all parameters in three diffusion models, 74 correlations were remarkable (**Figure 1**).

Among single model parameters, Mono-ADC_median_ and Mono-ADC_mean_ generated the best AUC in the positive and negative groups between ER (AUC = 0.766, *p*< 0.001) and PR (AUC = 0.735, *p*< 0.001), respectively. Meanwhile, DKI-Kapp_kurtosis_, IVIM-*D*_skewness_, and DKI-Dapp_skewness_ generated the best AUC in the positive and negative groups between HER2 (AUC = 0.632, *p* = 0.010), Ki-67 (AUC = 0.572, *p* = 0.049), and LNM (AUC = 0.603, *p* = 0.044), respectively. Regarding the differentiation of Luminal type *vs*. non-Luminal type, TN type *vs*. non-TN type, as well as HER2-positive type *vs*. non-HER2-positive type, the best AUC was derived from the Mono-ADC_mean_ (AUC = 0.785, *p*< 0.001), Mono-ADC_25th_ (AUC = 0.719, *p*< 0.001), and Mono-ADC_75th_ (AUC = 0.738, *p*< 0.001), respectively (**Table 5**).

**Table 5:** AUC of histogram metrics derived from mono, IVIM, and DKI models to predict molecular prognostic factors and subtypes.

	ADC	*D*	D*	*f*	Kapp	Dapp	ADC	*D*	D*	*f*	Kapp	Dapp
Metrics	ER-positive *vs*.ER-negative						PR-positive *vs*. PR-negative					
25th	0.752	0.687	0.513	0.523	0.737	**0.752**	0.705	0.648	0.511	0.508	0.692	0.679
Median	**0.766**	0.693	0.551	0.512	**0.753**	0.739	0.730	0.662	0.510	0.512	**0.707**	**0.704**
Mean	0.765	**0.702**	0.555	0.511	0.746	0.734	**0.735**	**0.671**	0.506	0.507	0.704	0.699
75th	0.750	0.689	**0.585**	**0.526**	0.742	0.717	0.728	0.665	0.534	0.531	0.698	0.701
Kurtosis	0.504	0.521	0.523	0.515	0.55	0.531	0.457	0.541	**0.559**	**0.533**	0.575	0.596
Skewness	0.518	0.537	0.535	0.514	0.538	0.507	0.516	0.549	0.556	0.527	0.558	0.514
Single model	0.766	0.706			0.755		0.735	0.671			0.719	
Three models	0.784						0.747					
Metrics	HER2-positive *vs*. HER2-negative						Ki-67-positive *vs*. Ki-67-negative					
25th	0.537	0.518	0.529	0.523	0.607	0.517	0.515	0.504	**0.539**	0.502	0.526	0.506
Median	0.565	0.541	0.500	0.540	0.581	0.557	0.518	0.508	0.524	0.557	**0.544**	0.505
Mean	0.584	0.552	0.503	0.559	0.581	0.566	0.524	0.516	0.522	**0.563**	0.535	0.509
75th	0.593	0.578	0.509	**0.575**	0.547	**0.602**	0.525	0.519	0.502	0.558	0.542	0.519
Kurtosis	**0.602**	**0.617**	0.527	0.504	**0.632**	0.593	0.514	0.503	0.501	0.539	0.527	0.538
Skewness	0.554	0.579	**0.545**	0.537	0.572	0.588	**0.569**	**0.572**	0.508	0.508	0.508	**0.560**
Single model	0.602	0.620			0.622		0.569	0.588			0.611	
Three models	0.659						0.630					
Metrics	LNM-positive *vs*. LNM-negative						Luminal *vs*. Non-Luminal					
25th	0.550	**0.540**	**0.563**	**0.577**	0.546	0.543	0.766	0.697	0.501	0.521	0.733	0.746
Median	0.534	0.514	0.541	0.568	0.544	0.521	0.781	0704	0.5234	0.526	**0.741**	**0.758**
Mean	0.546	0.507	0.543	0.548	0.554	0.535	**0.785**	**0.718**	0.528	0.527	0.738	0.752
75th	0.529	0.510	0.536	0.540	0.557	0.521	0.772	0.706	0.5151	**0.545**	0.737	0.741
Kurtosis	0.505	0.513	0.525	0.502	**0.559**	0.517	0.519	0.545	**0.556**	0.523	0.553	0.565
Skewness	**0.592**	0.501	0.514	0.514	0.525	**0.603**	0.503	0.529	0.561	0.529	0.562	0.532
Single model	0.592	0.580			0.617		0.785	0.722			0.773	
Three models	0.616						0.796					
Metrics	TN *vs*. Non-TN						HER2-positive *vs*. Non-HER2-positive					
25th	**0.719**	0.630	0.531	**0.541**	0.647	**0.704**	0.679	0.672	0.535	0.514	0.696	0.663
Median	0.701	0.625	0.525	0.513	0.673	0.684	0.726	0.689	**0.527**	0.526	0.696	0.707
Mean	0.697	**0.636**	0.532	0.529	0.662	0.678	0.737	0.698	0.508	0.511	**0.701**	0.705
75th	0.677	0.620	0.549	0.526	**0.685**	0.648	**0.738**	**0.699**	0.526	0.543	0.672	**0.723**
Kurtosis	0.508	0.531	0.573	0.514	0.513	0.503	0.541	0.512	0.505	0.522	0.572	0.612
Skewness	0.531	0.569	**0.577**	0.526	0.519	0.555	0.532	0.624	0.509	**0.579**	0.625	0.620
Single model	0.719	0.655			0.714		0.738	0.714			0.738	
Three models	0.736						0.747					

The best AUC of every diffusion parameter is shown in bold. ER, Estrogen receptor; PR, Progesterone receptor; HER2, Human epidermal growth factor receptor 2; LNM, Lymph nodes metastasis; TN, Triple-negative; D*, pseudo-diffusion coefficient.

Among single models, the DKI model generated the best AUC in the HER2-positive and HER2-negative groups (AUC = 0.622, *p* = 0.017), Ki-67-negative and Ki-67-positive groups (AUC = 0.611, *p* = 0.022), and LNM-positive and LNM-negative groups (AUC = 0.617, *p* = 0.022). The Mono model generated the best AUC in the ER-positive and ER-negative groups (AUC = 0.766, *p*< 0.001), PR-positive and PR-negative groups (AUC = 0.735, *p*< 0.001), Luminal type *vs*. non-Luminal type (AUC = 0.785, *p*< 0.001), as well as TN type *vs*. non-TN type (AUC = 0.719, *p*< 0.001). Both Mono and DKI models generated the best AUC in the HER2-positive type *vs*. non-HER2-positive type (AUC = 0.738, *p*< 0.001) (**Table 5**).

Regarding the differentiation of positive and negative groups between ER, PR, HER2, and Ki-67, the AUC of the combination of Mono, IVIM, and DKI resulted in the best discriminatory power compared with either model alone. The comparisons of Luminal type versus non-Luminal type, TN type versus non-TN type, and HER2-positive type versus non-HER2-positive type revealed that AUC considerably improved when the combination of Mono, IVIM, and DKI was used compared with either model alone (**Table 5**).

### Comparison of morphologic characteristics between the groups of molecular prognostic factors and subtypes

**Figure 2:**
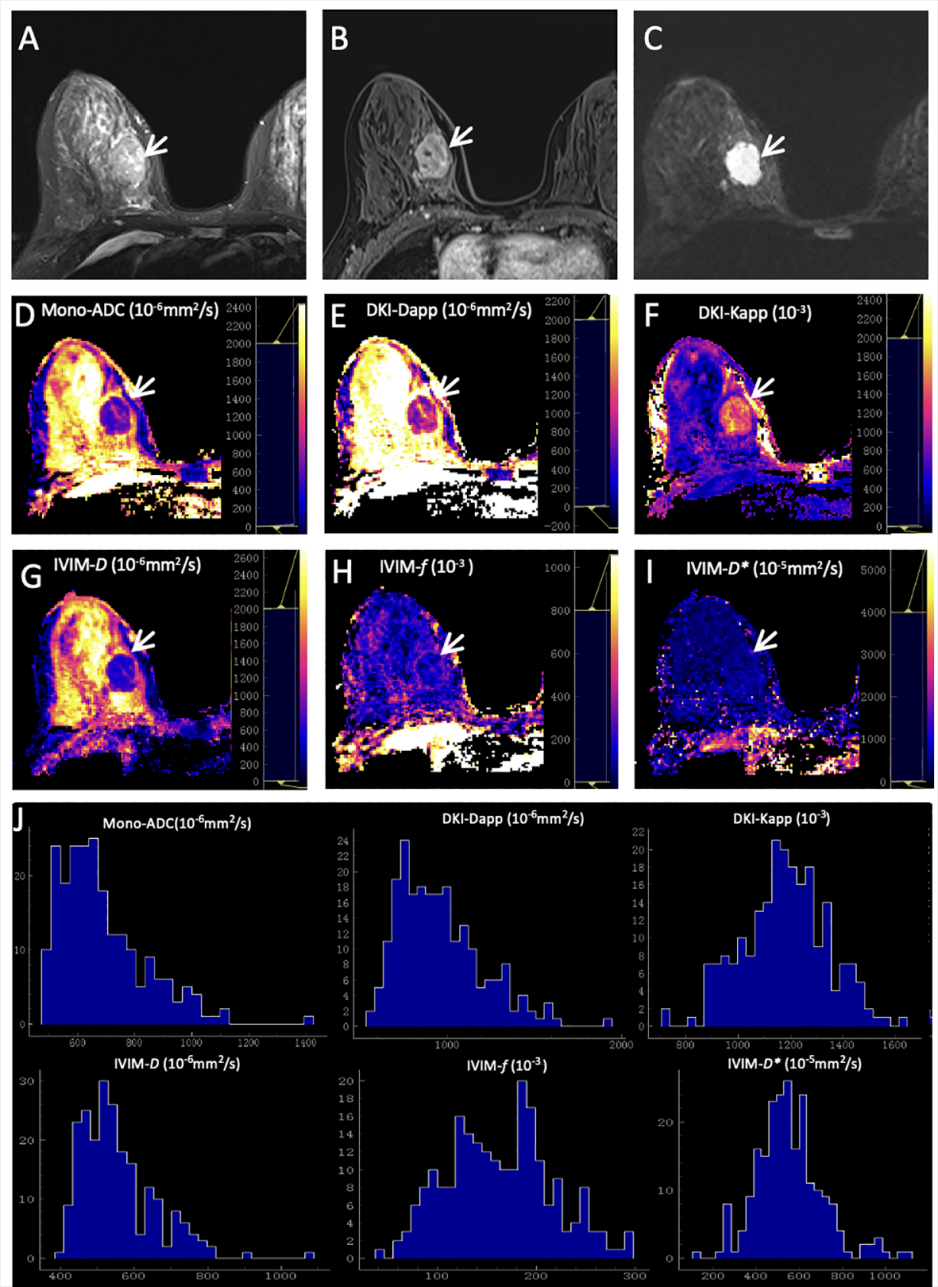
Representative images of a grade 2 invasive ductal carcinoma in the right breast of a 42-year-old woman. This tumor was with positive expression of estrogen receptor (95%) and progesterone receptor (20%), high Ki-67 (40%), and negative HER2 expression. **(A)** T2WI. **(B)** This mass is oval and with obvious enhancement (arrow) on axial DCE-MRI. **(C)** This mass shows oval shape, smooth margin, and homogeneous signal on DWI (b-value = 1000 mm^2^/s) (arrow). ADC **(D)**, Dapp **(E)**, Kapp **(F)**, *D* **(G)**, *f* **(H)**, and D* **(I)** maps and histograms of each map **(J)** are as shown.

**Figure 3:**
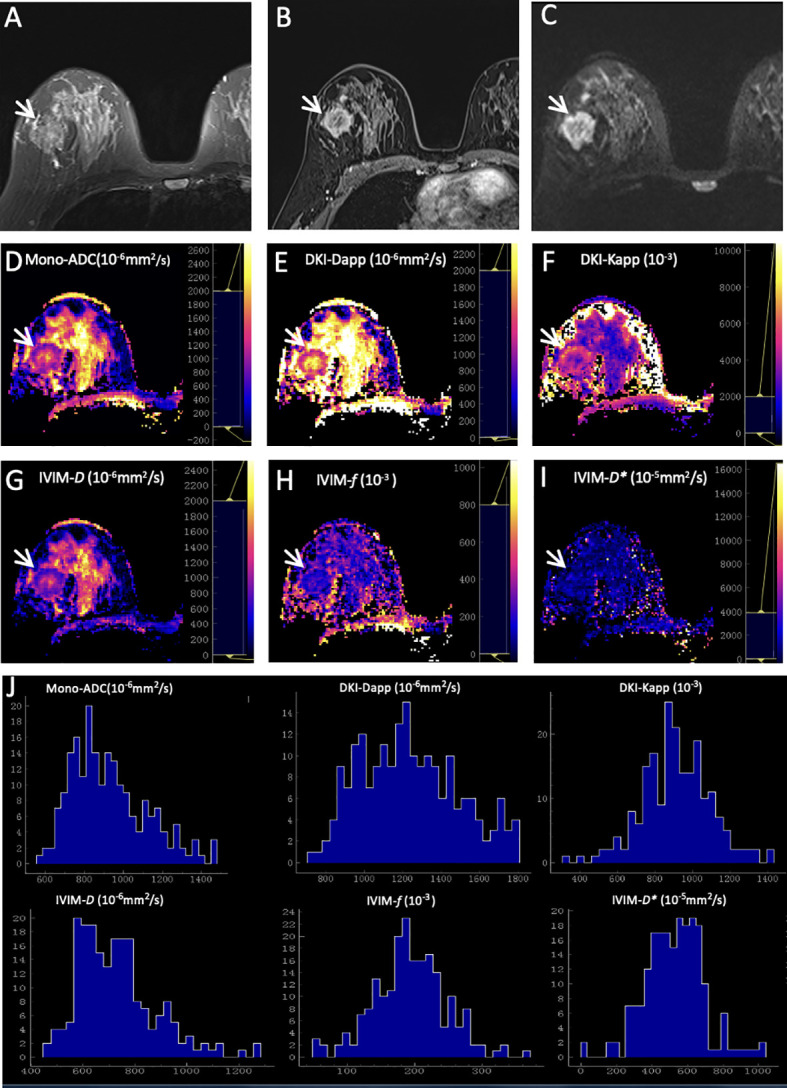
Representative images of a grade 3 invasive ductal carcinoma in the right breast of a 48-year-old woman. This tumor was with negative expression of estrogen receptor (0) and progesterone receptor (0), high Ki-67 (50%), and positive HER2 expression. **(A)** T2WI. **(B)** This mass is irregular and with obvious enhancement (arrow) on axial DCE-MRI. **(C)** This mass shows irregular shape, irregular margin, and heterogeneous signal on DWI (b-value = 1000 mm^2^/s) (arrow). ADC **(D)**, Dapp **(E)**, Kapp **(F)**, *D* **(G)**, *f* **(H)**, and D* **(I)** maps and histograms of each map **(J)** are as shown.

As summarized in **Table 6**, the results demonstrated that the margin of breast cancer had significant differences between the ER-positive and ER-negative groups (*p* = 0.002). No significant differences were observed in residual groups (all *p* > 0.00625). Two representative cases are shown in **Figures 2**; **3**.

**Table 6:** Magnetic resonance imaging morphological characteristics of molecular prognostic factors and subtypes.

		Max diameter		Shape				Margin				Internal signal			
groups			p-value	Round	Oval	Irregular	p-value	smooth	spiculated	irregular	p-value	homogeneous	heterogeneous	rim	p-value
ER	Positive	2.40 ± 0.96	0.269	11	37	32	0.092	52	13	15	0.002*	39	27	14	0.040
	Negative	2.59 ± 1.00		13	18	32		31	4	28		19	34	10	
PR	Positive	2.39 ± 0.90	0.209	11	34	31	0.253	49	9	18	0.187	21	34	12	0.097
	Negative	2.59 ± 1.01		13	21	33		34	8	25		9	11	14	
HER2	Positive	2.44 ± 0.90	0.788	10	16	22	0.544	27	6	15	0.953	20	19	9	0.840
	Negative	2.51 ± 0.99		14	39	42		56	11	28		38	42	15	
Ki-67	Positive	2.52 ± 0.94	0.409	17	30	32	0.214	51	7	21	0.186	30	34	15	0.668
	Negative	2.43 ± 0.97		7	25	32		32	10	22		28	27	9	
LNM	Positive	2.48 ± 0.95	0.992	6	18	25	0.443	26	6	17	0.652	20	21	8	0.994
	Negative	2.48 ± 0.96		12	39	35		57	11	26		38	40	16	
Luminal *vs*.		2.40 ± 0.93	0.230	12	16	29	0.106	55	13	18	0.010	42	30	14	0.034
Non-Luminal type		2.60 ± 0.10		5	16	10		28	4	25		16	31	10	
TN *vs*.		2.62 ± 0.99	0.301	6	10	16	0.636	16	3	13	0.332	8	19	5	0.074
Non-TN type		2.45 ± 0.95		18	45	48		67	14	30		50	42	19	
HER2-positive *vs.*		2.58 ± 1.03	0.680	6	6	13	0.230	12	1	12	0.069	8	12	5	0.626
Non-HER2-poitive type		2.46 ± 0.84		18	49	51		71	16	31		50	49	19	

*indicates that the correlation is significant at the level of 0.00625 (double-tailed). ER, Estrogen receptor; PR, Progesterone receptor; HER2, Human epidermal growth factor receptor 2; LNM, Lymph nodes metastasis; TN, Triple-negative.

## Discussion

In this study, we evaluated the correlation of Mono, IVIM, and DKI parameters with prognostic factors and molecular subtypes of breast cancer using histogram analysis. The Mono and DKI models yielded greater AUC to discriminate prognostic factors and molecular subtypes compared with the IVIM model. The AUC significantly improved when the combination of the three diffusion models was used compared with either model alone except for discriminating LNM-positive and negative. Additionally, the qualitative DWI analysis based on the morphologic characteristics could distinguish between ER-positive and -negative groups.

Previous studies have demonstrated the correlations of diffusion parameters derived from Mono, IVIM, and DKI models with breast cancer prognostic factors (18, 26, 28, 29). ER overexpression could inhibit angiogenesis to reduce perfusion contribution as well as increase cellularity to restrict water diffusion (11, 12, 29). Low perfusion contribution and high cellularity could both result in decreased histogram metrics of Mono-ADC, DKI-Dapp, and IVIM-*D* and increased histogram metrics of DKI-Kapp in the ER-positive group. The higher DKI-Kapp_mean_ in ER-positive tumors was consistent with the result of Yang et al. (16). Due to similarities in hormone receptor effects, PR-positive tumors also have same trend as ER-positive tumors. In our study, the histogram metrics of various diffusion parameters failed to reveal a remarkable difference between the statuses of HER2, Ki-67, and LNM. We speculated that this difference might be related to the inclusion of lesions, the selection of the *b* values, and the delineation of the ROI.

In terms of molecular subtypes, we analyzed them statistically in the form of binary classification. Previous studies demonstrated that IVIM-*D*_75th_ was lower in the Luminal type than in the HER2-positive type, and higher IVIM-*D* and lower IVIM-D* in Luminal A type than in the other subtypes (25, 30). These results were not entirely consistent with our study. Due to the Luminal type being defined as ER and/or PR positive, histogram metrics of Mono-ADC, IVIM-*D*, DKI-Dapp, and DKI-Kapp can be used to distinguish Luminal type from non-Luminal type, as similar to distinguishing ER and PR status. You et al. revealed that DKI-Kapp _entropy_ value could identify the HER2-positive type and non-HER2-positive type (20). Our study also showed DKI-Kapp histogram metrics, particularly mean, median, and 25th percentile, could differentiate HER2-positive type and non-HER2-positive type. Suo et al. have demonstrated higher Mono-ADC values in the TN subtype than in other subtypes (12); this tendency was also observed in our study with higher Mono-ADC, IVIM-D, and DKI-Dapp histogram metrics in the TN type than those in the non-TN type. The reason may be that the TN type shows a decrease in tumor cellularity with an associated increase in diffusion (31, 32). In summary, various diffusion parameters can quantify tissue cell density, perfusion contribution, and water motion *in vivo* and may serve as a potential biomarker for differentiating molecular subtypes.

Besides comparing individual parameters, the ROC of various models was also compared. The present study revealed that the AUC of the Mono or DKI model was higher than that of the IVIM model. That is, the Mono or DKI model was superior to the IVIM model in evaluating the correlations of prognostic factors and molecular subtypes of breast cancers. Yang et al. demonstrated that the DKI model was not superior to the Mono model in reflecting the prognostic information of breast cancer (16). Cho et al. demonstrated that the AUC of the IVIM model was higher than that of the Mono model, whereas Feng reported that the AUC of the IVIM model was lower than that of the Mono model (15, 17). The contradictory results might have resulted from the distinct choices of multi-*b* values and poor repeatability of multi-models. Therefore, the diagnostic value of the three models with various ranges of multi-*b* values needs further exploration.

Kul et al. reported that the morphology evaluated on DWI provided 83%-84% accuracy in distinguishing between benign and malignant lesions (33). However, Kang et al. reported that the specificity of the high-signal rim in DWI was higher than that of the ADC_mean_ value (80.6% *vs*. 63.9%) (34). Related studies include one by Cho, who showed that ER-positive tumor tends to show a not-circumscribed margin in mammography compared to ER-negative tumors (35). Different from our present study, the characteristic of smooth margin was more frequently observed in ER-positive tumors. Another study by Yuan et al, reported that the rate of burr sign in ER-positive in DCE-MRI was higher than that in negative groups (36). The trend was also observed in our study but was not significant. Although this study was a preliminary work, the morphologic characteristics assessed using SMS rs-EPI might provide a noninvasive tool for assessing the biologic characteristics and heterogeneity of breast cancers.

The present study had several limitations. First, the patient population was relatively small, and hence a selection bias might exist. Second, 2D ROI was manually drawn on the slice with the largest tumor diameter. This method did not reflect the overall tumor heterogeneity. Third, all MRI data were obtained in a single institution. Further studies are needed to verify the generalizability and reproducibility of our results.

In conclusion, the histogram metrics of multiparametric DWI and morphologic characteristics might be of use in providing prognostic information regarding breast cancer, thus potentially contributing to individualized treatment plans for patients with breast cancer.

## Data availability statement

The datasets presented in this article are not readily available because the datasets generated or analyzed during the study are available from the corresponding author on reasonable request. Requests to access the datasets should be directed to YQ, yanjinqin125@163.com.

## Author contributions

Conceptualization: YQ, FW, and TA. Data curation: YQ, CT, QH, LH. Formal analysis: YQ, QH, FW, and LH. Investigation: YQ, JY, and QH. Methodology: YQ, FW, CT, and MH. Project administration: TA. Software: TY and HZ. Supervision: TA. Visualization: YQ, FW, and MH. Writing - original draft: YQ and FW. Writing - review and editing: TA and TY. All authors contributed to the article and approved the submitted version.
